# Update on Temporal Lobe Epilepsy

**DOI:** 10.1155/2013/579487

**Published:** 2013-02-26

**Authors:** Seyed M. Mirsattari, Warren T. Blume

**Affiliations:** Discipline of Epileptology, The University of Western Ontario, London, ON, Canada N6A 5A5

Although the most common and best studied of focal epilepsies, a substantial proportion of patients with temporal lobe epilepsy (TLE) continue to have seizures despite medical therapy. Of these, surgery cannot be offered for most with bitemporal TLE while its effectiveness is limited in these and others because of memory and/or language concerns [1]. This up-to-date special edition contains a variety of valuable topics relative to TLE with the expectation that clues to unraveling this intractability will be found herein. 

Focal epilepsy occurs in 60% of patients with epilepsy and TLE is the most common of these (J. F. Téllez-Zenteno and L. Hernández-Ronquillo). These authors describe difficulties encountered by epidemiologists in identifying patients with TLE leading to a possible underestimation of this significant health care issue.

The description of TLE ictal semiology by Blair constitutes a useful introduction to this set of articles and may aid in distinguishing between mesial and neocortical temporal seizures. However, the several clinical features common to both mesial and neocortical TLE create a need for tests to distinguish these entities. As described by E. Bercovici et al., EEG and fMRI may aid in making this therapeutically important differentiation. Moreover, ictal semiology may vary considerably by age as documented for children by E. C. Wirrell et al. group and S. de Ribaupierre et al. and among the elderly by L. E. Morillo. 

S. Raghavendra et al. provide comprehensive evidence that interictal and ictal EEG remain essential contributors to localization of epileptogenesis. The thorough review of scalp and invasive EEG with a section on its automated analysis by M. Javidan helpfully complements the aforementioned review, providing the reader with an up-to-date picture of this topic. Early identification of interictal-to-ictal transition may be assisted by the measure of desynchronisationdescribed by J. Pastor et al.; high desynchronisation levels were found in MRI-normal mesial temporal epilepsy patients. That simultaneous EEG-fMRI recording may facilitate disclosure of the neurobiology of ictal and interictal epileptiform discharges, which is described by S. M. Mirsattari's group (Z. Wang).

Neuropsychologists play major roles in assessment of patients with temporal lobe epilepsy: (1) in localisation of dysfunction, thus aiding epileptogenesis localisation and (2) prediction of any postsurgical impairment of function in memory or language as reviewed by M. P. McAndrews and M. Cohn. 

Since the era of Wilder Penfield, a detailed pre- and postoperative memory evaluation has been considered requisite for temporal lobectomy consideration, especially when the left (language “dominant”) side is involved in epileptogenesis. The noninvasive fMRI, here described by C. Limotai and S. M. Mirsattari, may ultimately replace the Wada test to prognosticate the risk of significant postoperative memory decline. Fortunately, S. Oddo et al. found improvement in visual memory and executive function after temporal lobectomy among Argentinian patients.

A. Wang et al. describe the ability of functional MRI (fMRI) to map language networks in patients with intractable temporal lobe epilepsy and compares its capability in this assessment with more traditional tests such as Wada and cortical stimulation. The particular “challenges and solutions” of language mapping in children undergoing temporal lobectomy are described by S. de Ribaupierre et al. 

Temporal lobe neuroanatomy is a complex subject that perhaps is best comprehended by two approaches as presented in this volume. J. A. Kiernan, the anatomist, thoroughly depicts its multiple components, their terminology, and their many connections. 

As the neurosurgeon needs a complete and confident knowledge of the complex anatomy of the temporal lobe to perform an adequate resection with minimal to no neurological complications, the paper by B. Kucukyuruk et al. represents a “must read” for epilepsy surgeons in training. Knowledge of such intricate neuroanatomy and related physiology form the bases of the several resection approaches available to neurosurgeons as presented by F. Al-Otaibi et al. As described by D. Spencer and K. Burchiel, selective amygdalohippocampectomy is an alternative to the time-honoured standard temporal lobectomy. Reports of its effectiveness, covered by these authors, require scrutiny as it may be less so than its predecessor.

A variety of mental health abnormalities may be associated with or resemble epilepsy including: mood disorders, anxiety, and psychotic states (V. Beletsky and S. M. Mirsattari). Their symptoms, occasionally overlapping with those of epilepsy, may complicate both diagnosis and management. These authors cite Freud (1928) as questioning the accuracy of Dostoyevsky's self-diagnosis of a seizure disorder! Indicating the frequent cooccurrence of depression in patients with epilepsy, C. S. Garcia describes recent efforts in diagnosis and management of depression in those with epilepsy.

Integration of clinical findings of the patient with the several tests that may help to identify seizure origin determines surgical candidature; this process is well described by T. A. Valiante et al.

F. Al Sufiani and L. C. Ang have reviewed the several pathological abnormalities found in human temporal lobe resection specimens, including some newly recognised tumours; presumably, each contributes to epileptogenesis. The pathogenesis of mesial temporal sclerosis (MTS), the most common lesion found with temporal lobe seizures is more complex than first realised. Closely associated with a history of febrile seizures, the relationship may involve another temporal lobe lesion thus “dual pathology.” The illustrative case of cryptogenic status epilepticus leading to MTS presented by J. G. Boyd et al. further implicates prolonged seizures as a MTS aetiology. L. Carmant et al. comprehensively describe experimental models that may unravel the physiology of these associations. The natural history of TLE, reviewed by G. A. Shukla and N. Prasad, will likely provide further pathophysiological insights—particularly by scrutinising the latency period between an initial precipitating event and refractory TLE. This topic is further illustrated through an evidence-based approach as described by C. B. Josephson and B. Pohlmann-Eden. Although linkage analyses have thus far failed to disclose a gene related to TLE (A. Salzmann and A. Malafosse), the rapidly evolving field of human genetics may generate some future revelations in this regard.

As an estimated 80% of epilepsy patients live in underdeveloped countries (M. Z. Tahir et al.), the methods by which these authors established an epilepsy centre in Pakistan hold considerable interest. These include teleconferences, interactive teaching sessions, a nationwide workshop, public awareness events, and a visit to the proposed site by participating experts. 

*Seyed M. Mirsattari* *Seyed M. Mirsattari* *Warren T. Blume* *Warren T. Blume*
